# A Survey of Modulation of Gut Microbiota by Dietary Polyphenols

**DOI:** 10.1155/2015/850902

**Published:** 2015-02-22

**Authors:** Montserrat Dueñas, Irene Muñoz-González, Carolina Cueva, Ana Jiménez-Girón, Fernando Sánchez-Patán, Celestino Santos-Buelga, M. Victoria Moreno-Arribas, Begoña Bartolomé

**Affiliations:** ^1^Grupo de Investigación en Polifenoles, Unidad de Nutrición y Bromatología, Facultad de Farmacia, Universidad de Salamanca, Campus Miguel de Unamuno, 37007 Salamanca, Spain; ^2^Grupo de Biotecnología Enológica Aplicada, Instituto de Investigación en Ciencias de la Alimentación (CIAL), CSIC-UAM, Campus de Cantoblanco, C/Nicolás Cabrera 9, 28049 Madrid, Spain

## Abstract

Dietary polyphenols present in a broad range of plant foods have been related to beneficial health effects. This review aims to update the current information about the modulation of the gut microbiota by dietary phenolic compounds, from a perspective based on the experimental approaches used. After referring to general aspects of gut microbiota and dietary polyphenols, studies related to this topic are presented according to their experimental design: batch culture fermentations, gastrointestinal simulators, animal model studies, and human intervention studies. In general, studies evidence that dietary polyphenols may contribute to the maintenance of intestinal health by preserving the gut microbial balance through the stimulation of the growth of beneficial bacteria (i.e., lactobacilli and bifidobacteria) and the inhibition of pathogenic bacteria, exerting prebiotic-like effects. Combination of *in vitro* and *in vivo* models could help to understand the underlying mechanisms in the polyphenols-microbiota-host triangle and elucidate the implications of polyphenols on human health. From a technological point of view, supplementation with rich-polyphenolic stuffs (phenolic extracts, phenolic-enriched fractions, etc.) could be an effective option to improve health benefits of functional foods such as the case of dairy fermented foods.

## 1. Introduction

More and more studies confirm the importance of the gut microbiota in host health, including mental health. Gut bacteria not only help us to maximize the absorption of nutrients and energy, but also are essential in the body health status [1]. In particular, microbial infections and imbalances in the composition of the gut microbiota are associated with intestinal disorders such as chronic inflammatory bowel diseases and with other immune related disorders [2, 3]. Although genetic and environmental factors are main determinants of gut microbiota composition, it is well established that diet influences microbial fermentation and total bacteria in the intestine. In fact, interindividual variation in gut microbiota may, in part, reflect differences in dietary intake, although the response of the gut microbiota to dietary change can also differ among individuals [4].

**Figure 1:**
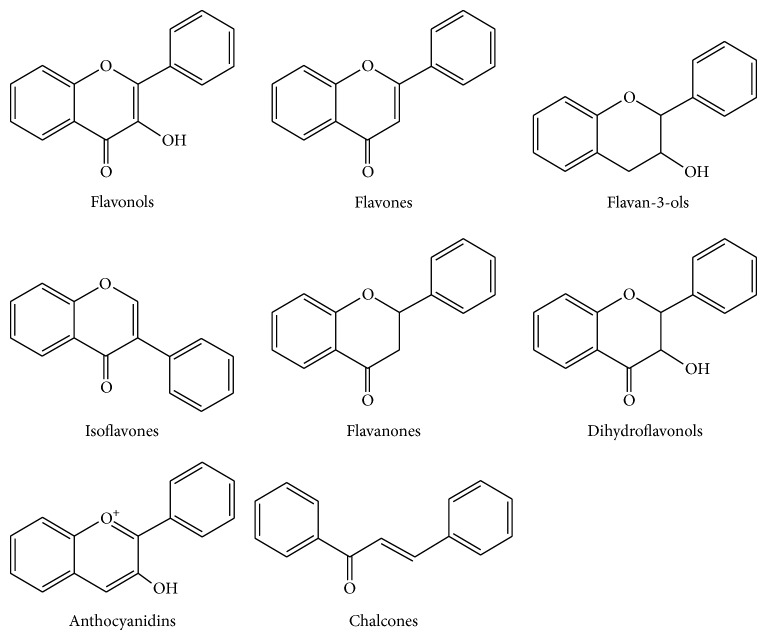
Common phenolic compounds in food.

Phenolic compounds or polyphenols are secondary metabolites with a widespread occurrence in the plant kingdom. In nature, polyphenols can be classified into two major groups: flavonoids and nonflavonoids. Among flavonoids, various groups can be distinguished according to the C-heterocycle structure: flavonols, flavones, flavan-3-ols, isoflavones, flavanones, dihydroflavonols, anthocyanidins, and chalcones (Figure 1). Nonflavonoid phenolics include phenolic acids, hydrolysable tannins, and stilbenes, among others. Polyphenols also form part of the human diet, being present in a broad range of commonly consumed fruits, vegetables, and plant-derived products such as cocoa, tea, or wine. A number of epidemiological studies have shown that the intake of diets rich in fruits and vegetables is inversely associated with the risk of various chronic diseases, such as coronary heart disease, specific cancers, and neurodegenerative disorders [5–7]. Indeed, a range of pharmacological effects have been demonstrated for different phenolic compounds—especially flavonoids—through* in vitro*,* ex vivo* and animal assays [8, 9]. However, health effects of these compounds depend on their bioavailability and, therefore, it is important to understand how they are absorbed, metabolized, and eliminated from the body, in order to ascertain their* in vivo* actions.

Modulation of gut microbiota by polyphenols has been a topic of increasing attention by the scientific community in the last years. Several studies have been carried out by different authors ranging from the simplest experimental approaches on the effect of polyphenols on the growth of isolated intestinal bacteria to complex approximations implying the whole fecal microbiota, either in fermentation experiments (batch cultures and continuous simulators) or through compositional analysis of animal and human fecal samples. The existing knowledge about relationships between polyphenols and gut microbiota has been object of many reviews from different perspectives. Thus, some authors have put their attention on the impact of food constituents (polyphenols included) in the gut microbiome [10, 11], while others have focused on the effects of dietary polyphenols on microbial modulation and their potential implications in human health [12–15]. Selma et al. [16] wrote probably the first review trying to put together the concepts of microbial degradation of polyphenols and modulation of gut microbiota by polyphenols and phenolic metabolites. This two-way interaction between phenolics and intestinal bacteria has been also reviewed focusing on wine [17] and tea polyphenols [18]. The development of improved biology and microbial techniques has allowed notable advances in the knowledge of the gut microbiota and their modulation by dietary components and hence polyphenols. The potential of the novel metabolomic approaches in the study of the impact of polyphenols on gut microbiome has been recently reviewed [19].

Being aware of all this previous reviewing work, we have aimed to update the available information about modulation of gut microbiota by dietary polyphenols with a perspective based on the experimental approaches used. After two general sections covering relevant aspects about gut microbiota (Section 2) and dietary polyphenols (Section 3), studies are presented according to their experimental design: batch culture fermentations (Section 4), gastrointestinal simulators (Section 5), animal model studies (Section 6), and human intervention studies (Section 7). Main findings and general conclusions generated from the different types of studies are finally discussed (Section 8).

## 2. Gut Microbiota Composition and Analysis

The human gut is the natural habitat of a large, diverse population and dynamics of microorganisms, mainly anaerobic bacteria, which have adapted to life on mucosal surfaces in the gut lumen. The acquisition of gut microbiota begins at birth and is strongly influenced by a range of factors that include host genetics, immunological factors, antibiotic usage, and also dietary habits [20]. The microbial content of the gastrointestinal tract changes along its length, ranging from a narrow diversity and low numbers of microbes in the stomach to a wide diversity and high numbers in the large intestine, which can reach 10^12^ CFU/mL [21]. Most of intestinal bacteria belong to phylum Firmicutes (including* Clostridium*,* Enterococcus*,* Lactobacillus*, and* Ruminococcus* genera) and Bacteroidetes (including* Prevotella* and* Bacteroides* genera) which constitute over 90% of known phylogenetic categories and dominate the distal gut microbiota [22]. Recently, a novel classification of microbiota into three predominant “enterotypes,” dominated by three different genera,* Bacteroides*,* Prevotella*, and* Ruminococcus*, has been suggested [23]. In this line, Wu et al. [24] demonstrated that long-term diet high in animal proteins and fats versus simple carbohydrates clustered the human subjects into the previously described enterotypes* Bacteroides* and* Prevotella*. However, there is a current debate if the enterotypes should be seen discontinuous or as a gradient [25]. But in any case, a common observation is that homeostasis and resilience are coupled to a highly diverse gut microbiota in healthy people, whereas inflammatory and metabolic disorders are linked to perturbations in the composition and/or functions of the gut microbiota [26].

Culture-based techniques employed to bacteria identification are fairly cheap, laborious, and time-consuming and gives a limited view of the diversity and dynamics of the gastrointestinal microbiota, with less than 30% of gut microbiota members having been cultured to date [27]. Since 1990s, the introduction of novel molecular biological procedures has made it possible to overcome some of these limitations with the use of culture-independent methods [28]. These procedures are based on sequence divergences of the small subunit ribosomal RNA (16S rRNA) and include techniques such as denaturing gradient gel electrophoresis (DGGE), terminal restriction fragment length polymorphism (T-RFLP), fluorescence* in situ* hybridization (FISH), quantitative polymerase chain reaction (qPCR), DNA microarrays, and next-generation sequencing (NGS) of the 16S rRNA gene or its amplicons [29]. NGS techniques have promoted the emergence of new, high-throughput technologies, such as genomics, metagenomics, transcriptomics, and metatranscriptomics. Metagenomics gives a more in-depth, unbiased microbial analysis beyond the group level and involves multiple species, besides showing shorter sequencing speed, extended read length, and lower costs [30]. However, the enormous amount of data generated becomes cumbersome to analyze and requires lots of dedicated time as well as expertise to manage [29].

In the context of polyphenol-microbiota interactions, the emerging high-throughput meta-genomic, transcriptomic, and proteomic approaches can be adopted to identify genes and micro-organisms involved in polyphenol (in)activation and conversion, to reconstruct metabolic pathways, and to monitor how microbial communities adjust their metabolic activities upon polyphenol exposure [30]. Application of these technologies to human fecal samples requires further investigation to determine how these samples reflect metabolism inside the gut and, ultimately, to improve the understanding of the impact of polyphenols on host health [12, 31].

## 3. Dietary Polyphenols

It has been estimated that 90–95% of dietary polyphenols are not absorbed in the small intestine and therefore reach the colon [32], although absorption and metabolism are largely influenced by their chemical structure. Most flavonoids are poorly absorbed from the small intestine and are highly metabolized in the large intestine. Isoflavones seem to be the best absorbed dietary flavonoids; catechins, flavanones, and flavonol glycosides are intermediate, whereas proanthocyanidins, flavan-3-ol gallates, and anthocyanins would be the worst absorbed [33].

The first step in the metabolism of flavonoids, with the exception of flavan-3-ols (i.e., catechins and proanthocyanidins), is likely to be deglycosylation before absorption in the small intestine. Hydrolysis of some flavonoid glycoside might already occur in the oral cavity, as both saliva and oral microbiota show *β*-glucosidase activity. But the mechanism most usually assumed for flavonoid deglycosylation is hydrolysis by lactase phlorizin hydrolase (LPH) in the brush-border of the small intestine epithelial cells [34, 35], so that the resulting aglycones would enter the enterocyte by passive diffusion. The resulting aglycone is rapidly biotransformed by phase II enzymes within the enterocyte and further in the liver, so that conjugated metabolites (i.e., glucuronides,* O*-mehtylethers, and/or sulphates) through the respective action of UDP-glucuronosyltransferase (UGT), catechol-*O*-methyltransferase (COMT), and sulphotransferases would be the circulating forms in the human body [36, 37].

Generally, a relevant fraction of dietary flavonoids is not absorbed in the small intestine and, together with the conjugated metabolites that returned to the intestinal lumen via enterohepatic circulation, reaches the large intestine where compounds are subjected to the action of the colonic microbiota. Intestinal bacteria show diverse deglycosylating activities, thus releasing aglycones that might be absorbed in a lesser extent and, more probably, degraded to simpler phenolic derivatives [38, 39]. Degradation of flavonoid aglycones by colonic microbiota involves ring-C cleavage and reactions affecting functional groups, such as dehydroxylation, demethylation, or decarboxylation [39]. Various hydroxylated aromatic compounds derived from the A-ring (e.g., phloroglucinol, 3,4-dihydroxybenzaldehyde, or 3,4-dihydroxytoluene) and phenolic acids derived from the B-ring have been reported as relevant products of the colonic transformation of flavonoids [40]. It has become evident that the beneficial effects attributed to dietary polyphenols appear to be due more to phenolic metabolites formed in the gastrointestinal tract, mainly derived from the action of gut bacteria, rather than to the original forms found in food [41].

In subsequent sections, main findings related to the modulation of gut microbiota by polyphenols are presented as obtained from different methodological approaches and microbial analysis techniques.

## 4. Studies Using Batch Culture Fermentations

Although* in vivo* human or animal intervention trials are physiologically most relevant to study both polyphenol metabolism and microbial modulation,* in vitro* tools have been designed to simulate intestinal conditions. In combination with* in vivo* trials,* in vitro* experiments may help to elucidate the extent bioconversion processes mediated by the host itself [42, 43]. The complexity of* in vitro* gut models is diverse, ranging from simple static models (batch culture fermentation) to advanced continuous models (gastrointestinal simulators).

Simple, static gut models, also known as batch-type cultures, are generally closed systems using sealed bottles or reactors containing suspensions of fecal material that are maintained under anaerobic conditions. They are relatively easy to operate and cost-effective, have a fair throughput, and allow for parallel screening. This model approach is primarily used to assess the stability of polyphenols in the presence of human-derived gut microbiota and to evaluate which environmental conditions favor or limit polyphenol bioconversion. However, these static gut models are only adequate for simulating short-term conditions in the gut; for assessment of long-term adaptations of the gut microbial community, more complex dynamic models are needed [12].

Table 1 reports different studies of modulation of gut microbiota by dietary polyphenols using batch-type cultures. Details about fermentation conditions (fecal concentration, polyphenol origin and dose, and incubation time) and microbial techniques used, and main effects on bacteria groups (growth enhancement, growth inhibition, or no effect) have been included. As general characteristics, fecal fermentations employed feces concentration ≤10% (w/v) and lasted 48 h maximum. Both pure phenolic compounds and phenolic-rich extracts were added to the fecal medium at a final concentration <10% (w/v), and changes in specific bacterial groups were mainly assessed by FISH analysis. A first relevant experiment using batch culture fermentation was carried out by Tzounis et al. [44] who found that the flavan-3-ol monomers [(−)-epicatechin and (+)-catechin] promoted the growth of* Clostridium coccoides*-*Eubacterium rectale* group, which is known to produce large amounts of butyrate, a short-chain fatty acid (SCFA) with anti-inflammatory, and antineoplasic properties; (+)-catechin also increased the growth of* Lactobacillus*-*Enterococcus* spp.,* Bifidobacterium* spp., and* Escherichia coli* but decreased the growth of* Clostridium histolyticum*. Also using standard compounds, Hidalgo et al. [45] found that anthocyanins (i.e., malvidin-3-glucoside and a mixture of anthocyanins) significantly enhanced the growth of* Lactobacillus-Enterococcus* spp. and* Bifidobacterium* spp. In addition, malvidin-3-glucoside showed a tendency to promote the growth of the* C. coccoides-E. rectale* group.

Similar results have been observed in batch culture fermentations with phenolic-rich extracts from different sources. Molan et al. [46] found that the addition of blueberry extracts to a mixture of fecal bacterial populations significantly increased the number of lactobacilli and bifidobacteria (Table 1). In the same line, Bialonska et al. [47] reported enhancement of the growth of total bacteria,* Bifidobacterium* spp., and* Lactobacillus-Enterococcus *spp. in response to a commercial extract of pomegranate, without influencing the* C. coccoides-E. rectale *and* C. histolyticum* groups (Table 1). Mandalari et al. [48] suggested a potential prebiotic effect for natural and blanched almond skins as these foodstuffs, in fermentations with fecal microbiota, significantly increased the populations of bifidobacteria and* C. coccoides-E. rectale* group and decreased the number of* C. hystolyticum* group. These authors related the possible prebiotic effect by almond skins not only to a high amount of dietary fibre, but also to some phenolic compounds such as ferulic acid, flavan-3-ols, and flavonols present in the almond skins [48]. Fogliano et al. [49] carried out an* in vitro* fermentation with a water-insoluble cocoa fraction in a three-stage continuous culture colonic model system. It was observed that this cocoa fraction presented prebiotic activity producing a significant increase in lactobacilli and bifidobacteria, as well as an increase in butyrate production. They concluded that the coexistence of fermentable polysaccharides and free flavanol monomers in cocoa, such as catechins, might be very effective in the modification of gut microbiota. Similar conclusions were drawn by Pozuelo et al. [50], who found a significant increase of the growth of* Lactobacillus reuteri* and* Lactobacillus acidophilus* in the presence of a grape antioxidant dietary fiber naturally obtained from red grapes. Our research group carried out several batch culture fermentations of two flavan-3-ol fractions with different degree of polymerisation and wine polyphenols, with fecal microbiota from different healthy volunteers [51, 52]. Both flavan-3-ol fractions promoted the growth of* Lactobacillus/Enterococcus *spp. and inhibited the* C. histolyticum* group during fermentation, although the effects were only statistically significant with the less polymerized fraction. Wine polyphenols only showed a slight inhibition in the* C. histolyticum* group, probably due to their lower content in flavan-3-ols.

Additionally, this type of fermentations has also been used to assess the contribution of certain probiotic strains to the colonic metabolism of polyphenols. In this sense, Barroso et al. [53] carried out fermentations of a red wine extract inoculated with human microbiota obtained from the colonic compartments of a dynamic simulator, in the presence and absence of the probiotic strain* L. plantarum* IFPL935. Microbial analysis by qPCR indicated that red wine polyphenols induced greater variations among* in vitro* batches harboring different colon-region (ascending colon, descending colon, and effluent) microbiota than those found when* L. plantarum* IFPL935 was added. Batches inoculated with microbiota from the ascending colon were shown to harbor the major proportion of saccharolytic bacteria (*Bacteroides*,* Bifidobacterium*, and* Prevotella*) whereas* Clostridium* groups were found in major numbers in the batches inoculated with microbiota simulating the distal regions [53] (Table 1).

## 5. Studies Using Human Gastrointestinal Simulators

In contrast to short-duration experiments with static gut models, longer-term experiments are required when the adaptation of the gut microbial community to dietary polyphenols needs to be assessed. To this end, dynamic* in vitro* gut models such as the “Reading” model [54], the Simulator of the Human Intestinal Microbial Ecosystem (SHIME), the TNO Intestinal Model 2 (TIM2) [55, 56], and the recent gastrointestinal simulator set up in our Institute (SIMGI) (unpublished work) have been developed where gut microbiota are cultured over a longer time frame (days to weeks) in one or multiple connected, pH controlled vessels representing different parts of the gastrointestinal tract.

As an example of the versatility and potential of human gastrointestinal simulators, Table 2 reports a series of studies about modulation of gut microbiota by polyphenols using the SHIME [57, 58]. This validated model comprises stomach and small intestinal sections for predigestion of food as well as vessels stimulating the ascending, transcending, and descending parts of the human colon, allowing assessment of changes in the different colonic areas that are very challenging to access in a human intervention. However, it should be underlined that this approach takes for granted that the extracts reach intact the colonic region, and no nutrient absorption is considered. The use of the SHIME to investigate the effects of a soy germ powder on the fermentative capacity of the simulated microbiota of the colon was the aim of a study carried out by De Boever et al. [57]. They observed that the addition of the soy germ powder in a 2-week treatment resulted into an overall increase of bacterial marker populations (Enterobacteriaceae, coliforms,* Lactobacillus* spp.,* Staphylococcus *spp., and* Clostridium *spp.), with a significant increase of 2 log10 units in the* Lactobacillus *spp. population. More recently, Kemperman et al. [31], using the twin-SHIME model, studied the influence of a bolus dose and a 2-week continuous administration of complex dietary polyphenols from black tea or red wine grape extracts on the colonic microbiota. The Twin-SHIME system, involving two models that run in parallel, was inoculated with the same fecal sample for direct comparison of the effect of the two polyphenol types. A combination of analyses including cultivation, PCR-denaturing gradient gel electrophoresis (DGGE), quantitative PCR, and high throughput pyrosequencing of the 16S ribosomal RNA gene was applied to characterize microbial community changes. This study showed that complex polyphenols in the context of a model system can modulate select members of the human gut microbiota, revealing novel targets potentially involved in polyphenol metabolism and/or resistant microbes to be further investigated for polyphenol metabolism or resistance mechanisms [31].

## 6. Animal Models Studies

It is widely assumed that preliminary evidence should be warranted in animal models before human intervention trials. Animal models contribute to better understanding the mechanisms and biological effects that could be likely to happen in the human body. The metabolism of polyphenols has been object of numerous animal studies (mostly in rodents), especially for their impact on metabolic disorders [58], but only a few of these studies have followed the dynamics and composition of the intestinal microbiota in association with polyphenol metabolites retrieved from the host. Caution is required in extrapolating results to humans because culture-independent comparisons have revealed that most bacterial genera and species found in mice are not seen in humans, although the distal gut microbiota of mice and humans harbors the same bacterial phyla [59]. In this section, studies performed in animals in order to assess the effects of polyphenols on the modulation of intestinal microbiota are summarized (Table 3). Experiments were mainly performed in rats, although other larger animals such as chicks, calves, or pigs have also been used. Gut microbial communities were evaluated by diverse methodologies including culture-based methods (plate count), DGGE, FISH, T-RFLP, qPCR, and metagenomic sequencing.

Animal studies performed in pigs [60] and in calves [61] demonstrated that tea polyphenols administration contributed to the improvement in the composition of the intestinal microbiota. Thus, the administration of tea polyphenols in pigs significantly increased the levels of lactobacilli whilst it diminished the levels of total bacteria and* Bacteroidaceae,* and a tendency to decrease in lecithinase positive clostridia including* C. perfringens* was also observed [60]. However, the reduction rate of* Bifidobacterium* spp. and* Lactobacillus* spp. was slow, while that of* C. perfringens* decreased faster in calves supplemented with the green tea extract [61].

Dolara et al. [62] showed that treatment with wine polyphenols in carcinogen-treated F344 rats was associated with a strong variation in the colonic microbiota, compared to the control-fed rats. Although the total bacterial counts and anaerobe/aerobe ratio of microorganisms in the feces from polyphenol-treated rats were similar to that from control rats, propionibacteria,* Bacteroides,* and Clostridia decreased while lactobacilli and bifidobacteria increased. Based on additional experiments, these authors concluded that reduction of oxidative damage, modulation of colonic flora, and variation in gene expression may be all connected in the action of wine polyphenols on the intestinal function and carcinogenesis.

In other study, rats fed with apple juice instead of drinking water showed more lactobacilli and bifidobacteria in fresh feces that differed from the controls by one-log10 colony forming units [63]. The same research group studied the effect of colloids isolated from apple pomace extraction juices on the intestinal microbiota in Wistar rats. An increase of* Bacteroidaceae* in almost one-log10 higher counts was observed in feces of rats fed with apple juice colloid than control rats [64]. Another animal experiment conducted to study the effect on intestinal microbiota, of the inclusion of grape pomace extracts in the diet of broiler chicks [65], found that, for the cecum, birds fed grape extracts had higher populations of* E. coli*,* Lactobacillus,* and* Enterococcus *species than birds in any other treatment group. These authors concluded that grape polyphenol-rich products modified the gut morphology and intestinal microbiota and increased the biodiversity degree of intestinal bacteria in broiler chicks.

Inclusion of condensed tannins (proanthocyanidins) extracted from* Acacia angustissima* on rat diet resulted in a shift in the predominant bacteria towards tannin-resistant Gram-negative Enterobacteriaceaeand* Bacteroides* species and reduced the number of Gram-positive* C. leptum* group [66]. Compatible results were obtained in an experiment with rats fed a proanthocyanidin-rich cocoa preparation [67], where the authors found a significant decrease in the proportion of* Bacteroides*,* Clostridium, *and* Staphylococcus* genera in the feces of cocoa-fed animals. Interestingly, reductions in* Clostridium *species were found to correlate with weight loss and decrease in body mass index.

Larrosa et al. [68] observed an increase in lactobacilli and bifidobacteria when resveratrol (3,5,4′-trihydroxy-*trans*-stilbene), which naturally occurs in grapes and grape-derived foodstuffs such as red wine, was administered to rats. After induction of colitis by dextran sulphate sodium, proliferation of both* E. coli* and enterobacteria was lower in rats treated with resveratrol than in control rats. This could be the result of an indirect effect of resveratrol-supplemented diet, which increased bifidobacteria and lactobacilli counts preventing the colonization and invasion of tissues by enterobacteria including* E. coli*.

Prebiotic activity of wild blackcurrant extracts observed in* in vitro* experiments was further confirmed in rats by Molan et al. [69]. A significant increase in the population size of lactobacilli and bifidobacteria was observed after daily administration of those extracts to rats. Similarly, a grape antioxidant dietary fibre preparation was found to increase the population of* Lactobacillus* spp. when fed to rats, whereas populations of* Bifidobacterium *spp. decreased and changes in* E. coli* and* Bacteroides vulgatus* counts were not significant [50].

Recently, Lacombe et al. [70] studied the composition and functional potential of the colon microbiota from rats fed a diet enriched in lowbush wild blueberries. Application of novel metagenomic techniques (Illumina shotgun sequencing) revealed a significant reduction in the relative abundance of the genera* Lactobacillus* and* Enterococcus* associated with wild blueberries intake. In addition, hierarchical analysis showed a significant increase in the relative abundance of the phylum Actinobacteria, the order Actinomycetales, and several novel genera under the family Bifidobacteriaceae and Coriobacteriaceae in the blueberries group. The authors indicated that although the microbiome of rats differs from humans, the applied model was a powerful tool to study population dynamics and related metabolic functions. Metagenomic studies can determine microbial community profiles, gene presence/absence and abundance, and functional repertoire; however, they can only infer an observed phenotype since a gene presence does not imply its expression or functionality [71].

## 7. Human Intervention Studies

Investigations involving the use of humans potentially provide the best models for studying the interactions of food components (e.g., polyphenols) with microbiota, although* in vivo* intervention trials hold inevitable practical and ethical limitations [12]. The use of cross-over designs where volunteers serve as their own control permits multilevel analysis schemes that increase power but requires a relevant number of volunteers to allow for statistically significant multivariate models [72]. Up to now, only a few studies have examined the* in vivo *impact of dietary polyphenols on the human gut microbiota, and most of them were focused on single polyphenol molecules and selected bacterial populations. A summary of human intervention studies about effects of polyphenols in the modulation of the intestinal microbiota is collected in Table 4. In these studies, the polyphenol dose used was much dependent on the type of food preparation and its concentration, normally ranging from 0.1 to 4%; the treatment time was also variable, from 10 days to 2 months, and the applied microbial techniques were diverse (plate count, DGGE, FISH, T-RFLP, and qPCR).

In a study with a reduced number of subjects (*n* = 8), Okubo et al. [73] reported a notably increase in the percentages of* Bifidobacterium *spp. in total fecal counts after an intervention with a product containing 70% of tea polyphenols. A significant decrease of* C. perfringens* and other* Clostridium *spp. was also observed during the intake period. However, in a crossover feeding study (number of volunteers not reported) that investigated the effects of black tea drinking on hypercholesterolemic volunteers, Mai et al. [74] found that although specific bacterial groups were not affected, the total amount of bacteria significantly decreased, highlighting large interindividual variations. More recently, an intervention study (*n* = 10) by Jin et al. [75] confirmed an overall tendency for the proportion of bifidobacteria to increase because of green tea consumption, even though there were interindividual differences in the* Bifidobacterium* species.

Yamakoshi et al. [76] showed that administration of a proanthocyanidin-rich extract from grape seeds to healthy volunteers (*n* = 9) significantly increased the fecal number of* Bifidobacterium*, whereas the number of putrefactive bacteria such as enterobacteria tended to decrease. The interaction between proanthocyanidins and intestinal bacteria was also confirmed in a randomized, double-blind, crossover, and controlled intervention study (*n* = 22) ingesting two cocoa drinks exhibiting low and high polyphenol content [77]. Compared with the consumption of the low-flavan-3-ol cocoa drink, the daily consumption of the high-flavan-3-ol cocoa drink significantly increased the bifidobacteria and lactobacilli populations but significantly decreased clostridia counts.

Queipo-Ortuño et al. [78] performed a randomized, crossover, and controlled trial (*n* = 10) consisting of the intake of red wine, dealcoholized red wine, and gin over three consecutive periods. After the red wine period, the bacterial concentrations of proteobacteria, fusobacteria, Firmicutes, and Bacteroidetes markedly increased compared with the washout period; significant increases in the number of* Bifidobacterium* spp. and* Prevotella* spp. were also observed. However,* Lactobacillus* spp.,* Clostridium *spp., and* C. histolyticum* group concentrations remained unchanged throughout the study.

In a small-scale observational study (*n* = 8), Shinohara et al. [79] found that the number of bifidobacteria in feces significantly increased during apple intake and the numbers of* Lactobacillus *spp.,* Streptococcus* spp., and* Enterococcus* spp. tended to increase. On the contrary, enterobacteria and lecithinase-positive clostridia, including* C. perfringens* and* Pseudomonas *species, tended to decrease. However, that study did not use culture-independent microbiology techniques and suffered from the lack of a control group. Also in relation to fruits, another small human intervention study (*n* = 10) with raspberry puree [80] did not observe statistically significant alterations in the profile of colonic bacteria, probably due to high interindividual variation in fecal bacteria, although the profiles of microbial metabolites of raspberry polyphenols varied greatly between individuals, indicating that the type of gut microbiota affects catabolite profiles released by bacteria in the colon. This lack of effect on the intestinal microbiota after the intake of raspberry puree might also be due to the short duration of the treatment, as well as the techniques employed to quantify the intestinal microbiota.

Vendrame et al. [81] studied the potential prebiotic activity of a drink elaborated from wild blueberries especially rich in anthocyanins, in a small intervention trial (*n* = 15). A significant increase in* Bifidobacterium* spp. and* L. acidophilus* group was detected, while no significant differences were observed for* Bacteroides *spp.,* Prevotella* spp.,* Enterococcus *spp., and* C. coccoides*. In a further paper of the same group [82], seven different intragenus bifidobacteria taxonomic clusters that were among the most common and abundant bifidobacteria species inhabiting the human gut were targeted in the same samples. It was found that* B. adolescentis*,* B. breve*,* B. catenulatum/pseudocatenulatum,* and* B. longum *subsp.* longum* were always present in the group of subjects enrolled, whereas* B. bifidum* and* B. longum *subsp.* infantis* were not. In spite of the large interindividual variability, a significant increase of* B. longum *subsp.* infantis* cell concentration was observed in the feces of volunteers after the wild blueberry drink treatment, which was attributed to the presence of prebiotic (bifidogenic) molecules from blueberries, possibly fibers and glycosylated anthocyanins.

In a study with postmenopausal women (*n* = 39), Clavel et al. [83] found that isoflavone supplementation stimulated dominant microorganisms of the* C. coccoides-E. rectale* cluster,* Lactobacillus-Enterococcus* group,* Faecalibacterium prausnitzii* subgroup, and* Bifidobacterium* genus. It was also suggested that the concentration of* C. coccoides-E. rectale* cluster was related to women capacity to excrete equol, an intestinal metabolite from daidzein. In two intervention studies with whole grain breakfast cereals from wheat (*n* = 31) and maize (*n* = 32) [84, 85], the ingestion of both products resulted in significant increases in fecal bifidobacteria and/or lactobacilli without changing the relative abundance of other dominant members of the gut microbiota. Little or no changes were observed in the numbers of total bacteria,* Bacteroides* spp.,* C. histolyticum/perfringens* group, and* Acetobacterium* spp. present in the feces. However, as whole grains are good sources of dietary fiber, it is difficult to ascribe the observed effects only to the phenolic compounds present in these foods. In this respect, Cuervo et al. [86] have recently studied the correlations between the intake of fiber and polyphenols from diet and fecal microbiota composition in a cohort of apparently healthy subjects. Results showed that the intake of soluble pectins and flavanones from oranges presented a negative correlation with the levels of* B. coccoides* and* C. leptum*. By contrast, the intake of white bread, providing hemicellulose and resistant starch, was directly correlated with* Lactobacillus*.

Finally, another human trial (*n* = 16) carried out by Jaquet et al. [87] assessed the impact of a moderate consumption of instant coffee on the general composition of the human intestinal bacterial population. Coffee beverages contain significant amounts of soluble fibre (mainly galactomannans and arabinogalactan-proteins) and phenolic compounds (chlorogenic acids), which are well utilised by the human fecal microbiota. It was observed that although fecal profiles of the dominant microbiota were not significantly affected after the consumption of the coffee, the population of* Bifidobacterium* spp. increased, being the largest increase observed for those volunteers showing the lowest initial bifidobacteria levels.

## 8. Conclusions

This review has tried to summarize the current knowledge in relation to the phenolic metabolism by gut microbiota and the modulation of the gut microbiota by phenolic compounds and polyphenol-rich dietary sources. There are evidences that the beneficial effects attribute to dietary polyphenols depend on their biotransformation by the gut microbiota. Therefore, it is important to investigate the bacterial species implicated in the metabolism of dietary polyphenols, and further research is still needed in relation to the resultant microbial metabolites to ascertain their mechanisms of action. On the other hand, a great number of* in vitro* and* in vivo* (in animals and humans) studies showing the influence of dietary polyphenols on gut-inhabiting bacteria have been published in recent years. Although* in vitro* assays facilitate experimentation, caution must be taken in extrapolating results to* in vivo* situation, as many factors are acting upon this process. In general, in both* in vitro* and* in vivo* studies, polyphenols or polyphenol-rich sources have shown to influence the relative abundance of different bacterial groups within the gut microbiota, reducing numbers of potential pathogens, including* C. perfringens* and* C. histolyticum*, and certain Gram-negative* Bacteroides* spp. and enhancing mainly beneficial Clostridia, bifidobacteria and lactobacilli. A better understanding of the interaction between dietary polyphenols and gut microbiota through the emerging advances in high-throughput meta-genomic, transcriptomic, and proteomic approaches, would be essential in order to identify genes and micro-organisms involved in polyphenol (in)activation and conversion and thus, to elucidate the implications of diet on the modulation of microbiota for delivering health benefits.

Functional foods are considered to enhance the protective effects against diseases derived from some food components. In the last decades, dairy fermented foods have probably been one of the most-developed functional products and have deserved intensive research. In this expansion, dairy fermented foods have been supplemented with fruits, cereals, and other stuffs of plant origin, all of which represent a high percentage of the current market of the dairy industry. These products have a healthy appeal, which attracts consumers. Thus, fruit juices/concentrates, and prepared fruits (in the form of pieces, pulp, and even flour) have been successfully incorporated in dairy fermented foods as sources of prebiotic fibers and phytochemicals. Among these phytochemicals present in plant-derived foods, polyphenols have gained much interest due to their diverse potential beneficial effects in human health. The supplementation of dairy fermented products with rich-polyphenolic stuffs (phenolic extracts, phenolic-enriched fractions, etc.) seems to be an effective technological option to improve the benefits of these products in the balance of the intestinal microbiota, due not only to the action of the probiotics but also to the potential modulation effects exerted by polyphenols, as it has been described in this review. Further research in this area will aim to accomplish the benefits of both probiotic strains and polyphenols in relation to gut health.

## Figures and Tables

**Table 1:** Studies using batch culture fermentation.

Reference	Fecal concentration	Phenolic compound/food	Dose	Time of incubation	Microbial technique	Growth enhancement	Growth inhibition	No effect
Tzounis et al. (2008) [44]	10%, w/v	(+)-catechin	150 mg/L, 1000 mg/L	<48 h	FISH	*Lactobacillus-Enterococcus* spp. *Bifidobacterium *spp. *C. coccoides-E. rectale* group *E. coli *	*C. histolyticum* group	

Molan et al. (2009) [46]	0.1%, v/v	Blueberry extracts	5, 10 and 25%	48 h	FISH	Lactobacilli Bifidobacteria		

Bialonska et al. (2010) [47]	10%, w/v	Pomegranate extract and punicalagin	10%	48 h	FISH	Total bacteria *Bifidobacterium* spp. *Lactobacillus*-*Enterococcus* spp.		*C. coccoides-E. rectale* group *C. histolyticum* group

Mandalari et al. (2010) [48]	10%, w/v	Almond skins	1%, w/v predigested almond skins	<24 h	FISH	Bifidobacteria *C. coccoides-E*. *rectale* group	*C. histolyticum* group	

Fogliano et al. (2011) [49]	5%, w/v	Water-insoluble cocoa fraction	1%, w/v	36 h	FISH	Bifidobacteria Lactobacilli		

Cueva et al. (2013) [51]	10%, w/v	Grape seed extract fractions	300–450 mg/L	<48 h	FISH	*Lactobacillus-Enterococcus* spp.	*C. histolyticum* group	

Hidalgo et al. (2012) [45]	10%, w/v	Malvidin-3-*O*-glucoside Anthocyanidins mixture	20 mg/L and 200 mg/L 4850 mg/L and 48500 mg/L	<24 h	FISH	*Lactobacillus-Enterococcus* spp. *Bifidobacterium* spp. *C. coccoides*-*E. rectale* group		

Sánchez-Patán et al. (2012) [52]	1% w/v	Red wine extract	600 mg/L	48 h	FISH		*C. histolyticum* group	*Lactobacillus-Enterococcus* spp.

Barroso et al. (2013) [53]		Red wine extract	500 mg/L	48 h	qPCR	*Lactobacillus* spp. *Bifidobacterium* spp. *Bacteroides* spp. *Ruminococcus* spp.		

**Table 2:** Studies using the gastrointestinal simulators (i.e., SHIME).

Reference	Simulator	Phenolic compound/food	Dose	Time	Microbial technique	Population increase	Population decrease
De Boever et al. (2000) [57]	SHIME	Soy germ powder	2.5 g/day	2 weeks	Plate count	Enterobacteriaceae Coliforms *Lactobacillus* spp. *Staphylococcus* spp. *Clostridium* spp.	

Kemperman et al. (2013) [31]	Twin-SHIME	Black tea extract	3 × daily dosing (1000 mg polyphenols as total daily dose)	2 weeks	Plate count qPCR PCR-DGGE pyrosequencing	*Klebsiella* spp. Enterococci *Akkermansia* spp.	Bifidobacteria *Blautia coccoides* *Anaeroglobus *spp. *Victivallis* spp.

Kemperman et al. (2013) [31]	Twin-SHIME	Red wine-grape extract	3 × daily dosing (1000 mg polyphenols as total daily dose)	2 weeks	Plate count qPCR PCR-DGGE pyrosequencing	*Klebsiella* spp. *Alistipes* spp. *Cloacibacillus * spp. *Victivallis* spp. *Akkermansia* spp.	Bifidobacteria *Blautia coccoides* group *Anaeroglobus* spp. *Subdoligranulum *spp. *Bacteroides *

**Table 3:** Animal model studies.

Reference	Animal	Phenolic compound/food	Dose	Treatment duration	Microbial technique	Population increase	Population decrease
Hara et al. (1995) [60]	Pigs	Tea polyphenols	0.2% (free access)	2 weeks	Plate count	Lactobacilli	Total bacteria *Bacteroidaceae* *C. perfringens *

Ishihara et al. (2001) [61]	Calves	Green tea extracts	1.5 g/day	4 weeks	Plate count		*Bifidobacterium* spp. *Lactobacillus* spp. *C. perfringens *

Smith and Mackie (2004) [66]	Rats	Proantocyanidins extracted from *Acacia angustissima *	0.7% (low tannin diet) and 2.0% (high tannin diet)	3.5 weeks treatment + 3.5 weeks washout	PCR-DGGE Dot blot hybridization	*Bacteroides fragilis *group *Bacteroides-Prevotella-Porphyromonas* group Enterobacteriaceae	*C. leptum* group

Dolara et al. (2005) [62]	Rats	Red wine polyphenols powder	50 mg/kg	16 weeks	Plate count	Lactobacilli Bifidobacteria	Propionibacteria *Bacteroides* Clostridia

Sembries et al. (2006) [63]	Rats	Apple juice	free access	4 weeks	Plate count	Lactobacilli Bifidobacteria	

Sembries et al. (2003) [64]	Rats	Apple pomace juice colloid	5% suppl. diet (free access)	6 weeks	Plate count FISH	*Bacteroidaceae *	

Larrosa et al. (2009) [68]	Rats	Resveratrol	1 mg/kg/day	25 days	Plate count	Lactobacilli Bifidobacteria	

Molan et al. (2010) [69]	Rats	Blackcurrant extracts (leaf or berry)	3 times/week: (i) 30 mg/kg (leaf) (ii) 13.4 mg/kg (berry)	4 weeks	FISH	Lactobacilli (berry extract) Bifidobacteria (leaf and berry extracts)	

Viveros et al. (2011) [65]	Broiler chicks	Grape pomace concentrate (GPC) Grape seed extract (GSE)	60 g/kg diet (GPC) 7.2 g/kg diet (GSE) (free access)	21 days	Plate count T-RFLP	*E. coli* *Enterococcus *spp. *Lactobacillus* spp.	

Lacombe et al. (2013) [70]	Rats	Lowbush wild blueberries	20 g feed/day (eq. 24 ± 5.2 mg anthocyanin/day)	6 weeks	Metagenomic sequencing	Thermonospora spp. *Corynebacteria *spp. *Slackia* spp.	*Lactobacillus* spp. *Enterococcus *spp.

**Table 4:** Human intervention studies.

Reference	Volunteer number	Phenolic compound/food	Dose	Treatment duration	Microbial technique	Population increase	Population decrease	No effect
Okubo et al. (1994) [73]	8	Green tea (Sunphenon)	0.4 g/3 times per day	4 weeks	Plate count		*C. perfringens* *Clostridium* spp.	

Yamakoshi et al. (2001) [76]	9	Proantocyanidin-rich extract from grape seeds	0.5 g/day	6 weeks	Plate count	*Bifidobacterium* spp.	Enterobacteriaceae	

Mai et al. (2004) [74]	15	Black tea	700 mg tea solids/5 times per day	21 days	FISH DGGE		Total bacteria	No changes

Clavel et al. (2005) [83]	39	Isoflavones	100 mg/day	2 months	TTGE FISH	*C. coccoides-E. rectale* group *Bifidobacterium* spp. *Lactobacillus-Enterococcus* spp. *Faecalibacterium prausnitzii *subgroup		

Costabile et al. (2008) [84]	31	Whole grain wheat cereals	48 g/day	3 weeks	FISH	Bifidobacteria Lactobacilli		Total bacteria *Bacteroides* spp. *C. histolyticum/perfringens* group *Acetobacterium* spp.

Jaquet et al. (2009) [87]	16	Coffee	3 cups/day	3 weeks	FISH DGGE	*Bifidobacterium* spp.		

Carvalho-Wells et al. (2010) [85]	32	Whole grain maize cereals	48 g/day	3 weeks	FISH	Bifidobacteria		Total bacteria *Bacteroides* spp. *C. histolyticum/perfringens* group *Acetobacterium* spp.

Gill et al. (2010) [80]	10	Raspberry puree	20 g/day	4 days	PCR-DGGE			No changes in the profile of colonic bacteria

Shinohara et al. (2010) [79]	8	Apples	2 apples/day	2 weeks	Plate count	*Lactobacillus *spp. *Streptococcus *spp. *Enterococcus* spp.	Enterobacteriaceae lecithinase-positive clostridia including *C. perfringens*, *Pseudomonas* spp.	

Tzounis et al. (2011) [77]	22	Cocoa flavanol	494 mg/day 29 mg/day	4 weeks	FISH	*Bifidobacterium* spp. *Lactobacillus* spp.	*C. histolyticum/perfringens* group	

Vendrame et al. (2011) [81]	15	Wild blueberry drink	25 g wild blueberries/day	6 weeks	qPCR	*Bifidobacterium *spp. *L. acidophilus *		*Bacteroides* spp. *Prevotella* spp. *Enterococcus* spp. *C. coccoides *

Queipo-Ortuño et al. (2012) [78]	10	Red wine	272 mL/day	20 days	qPCR	*Enterococcus* spp. *Prevotella* spp. *Bacteroides Bifidobacterium* spp. *Bacteroides uniformis* *Eggerthella lenta* *Blautia coccoides-E. rectale* group	*Clostridium *spp. *C. histolyticum group *	Actinobacteria

Jin et al. (2012) [75]	10	Green tea	1000 mL/day	10 days	T-RFLP qPCR	*Bifidobacterium* spp.		

Guglielmetti et al. (2013) [82]	15	Wild blueberries drink	25 g wild blueberries/day	6 weeks	qPCR	*B. longum *subsp. *infantis *		

Cuervo et al. (2014) [86]	38	Dairy products Fruits Vegetables Cereals		Food intake was recorded using an annual food frequency questionnaire	qPCR	*Lactobacillus *	*B. coccoides* *C. leptum *	
